# Case report: a step-wise management of concurrent presentation of congenital single lung and aberrant right subclavian artery in an infant girl

**DOI:** 10.1186/s13019-021-01520-z

**Published:** 2021-05-25

**Authors:** Keon Young Park, Kevin C. Janek, Joshua L. Hermsen, Petros V. Anagnostopoulos, Hau D. Le

**Affiliations:** 1 Department of Surgery, University of California San Francisco, 400 Parnassus Avenue, San Francisco, CA USA; 2 Division of Pediatric Surgery, Department of Surgery, American Family Children’s Hospital, University of Wisconsin School of Medicine and Public Health, 600 Highland Avenue, MC 7375, Madison, WI 53792 USA; 3 Division of Cardiothoracic Surgery, Department of Surgery, American Family Children’s Hospital, University of Wisconsin School of Medicine and Public Health, Madison, WI USA

**Keywords:** Case report, Congenital single lung, Dysphagia lusoria, Congenital thoracic vascular anomaly, Tracheomalacia

## Abstract

**Introduction:**

Congenital single lung (CSL) is a rare condition, and symptomatic patients often present with respiratory distress or recurrent respiratory infection due to mediastinal shift causing vascular or airway compression. Aberrant right subclavian artery (ARSA) is another rare congenital anomality that can lead to tracheal or esophageal compressions. There is only one other case of concurrent presentation of CSL and ARSA reported, which presented unique challenge in surgical management of our patient. Here we present a step-wise, multidisciplinary approach to manage symptomatic CSL and ARSA.

**Case presentation:**

An infant girl with a prenatal diagnosis of CSL developed worsening stridor and several episodes of respiratory illnesses at 11 months old. Cross-sectional imaging and bronchoscopic evaluation showed moderate to severe distal tracheomalacia with anterior and posterior tracheal compression resulting from severe mediastinal rotation secondary to right-sided CSL. It was determined that her tracheal compression was mainly caused by her aortic arch wrapping around the trachea, with possible additional posterior compression of the esophagus by the ARSA. She first underwent intrathoracic tissue expander placement, which resulted in immediate improvement of tracheal compression. Two days later, she developed symptoms of dysphagia lusoria due to increased posterior compression of her esophagus by the ARSA. She underwent transposition of ARSA to the right common carotid with immediate resolution of dysphagia lusoria. As the patient grew, additional saline was added to the tissue expander due to recurrence in compressive symptoms.

**Conclusions:**

Concurrent presentation of CSL and ARSA is extremely rare. Asymptomatic CSL and ARSA do not require surgical interventions. However, if symptomatic, it is crucial to involve a multidisciplinary team for surgical planning and to take a step-wise approach as we were able to recognize and address both tracheomalacia and dysphagia lusoria in our patient promptly.

## Introduction

Congenital single lung (CSL) is a rare condition, affecting about 1–3.4 per 100,000 live births [1]. Although CSL can be unilateral or bilateral, right-sided CSL is more likely to have mediastinal shift causing compression of vascular structures or airway, causing recurrent respiratory infection or respiratory distress during childhood [2]. If not managed promptly, the mortality can be up to 2% during the first year of life and up to 50% within the first 5 years of life [3].

Aberrant right subclavian artery (ARSA) originates on the left and beyond the origin of the left subclavian artery and occurs in 0.5–2% of the population. Typically, the artery courses behind the esophagus (80% of the incidence), between the esophagus and trachea (15%), or in front of the trachea (5%) [4]. Although 95% of cases of ARSA are asymptomatic, it can cause airway compromise in children due to the compressible nature of the pediatric trachea. Dysphagia can occur, and usually presents in adulthood due to aneurysmal dilation of the arterial origin (Kommerell’s diverticulum) or calcification of the vessel.

Here we present a unique challenge of treating an infant girl with concurrent presentation of initially asymptomatic CSL and ARSA. She developed anterior tracheal compression at 11 months old, which was corrected by placement of an extrapleural intrathoracic tissue expander. This intervention led to dysphagia lusoria due to increased posterior compression of her esophagus by ARSA requiring an additional surgical intervention.

## Case presentation

A 2.8 kg female born at 38 weeks and 5 days gestation age to a healthy 29-year-old mother was diagnosed prenatally with left congenital single lung (CSL) on routine ultrasound. After birth, she was admitted to the neonatal intensive care unit for non-invasive respiratory support. Her respiratory status improved by 12 h of life and she was able to be weaned off to room air. Cross-sectional imaging revealed absence of the right lung and right pulmonary artery, presence of a right mainstem bronchus remnant, and significant rightward displacement of her heart into right chest. She was found to have an H-type tracheoesophageal fistula (TEF) and intestinal malrotation. The H-type TEF was repaired via bronchoscopy and her malrotation was repaired laparoscopically without complications. She was discharged home on day of life (DOL) 21.

**Fig. 1:**
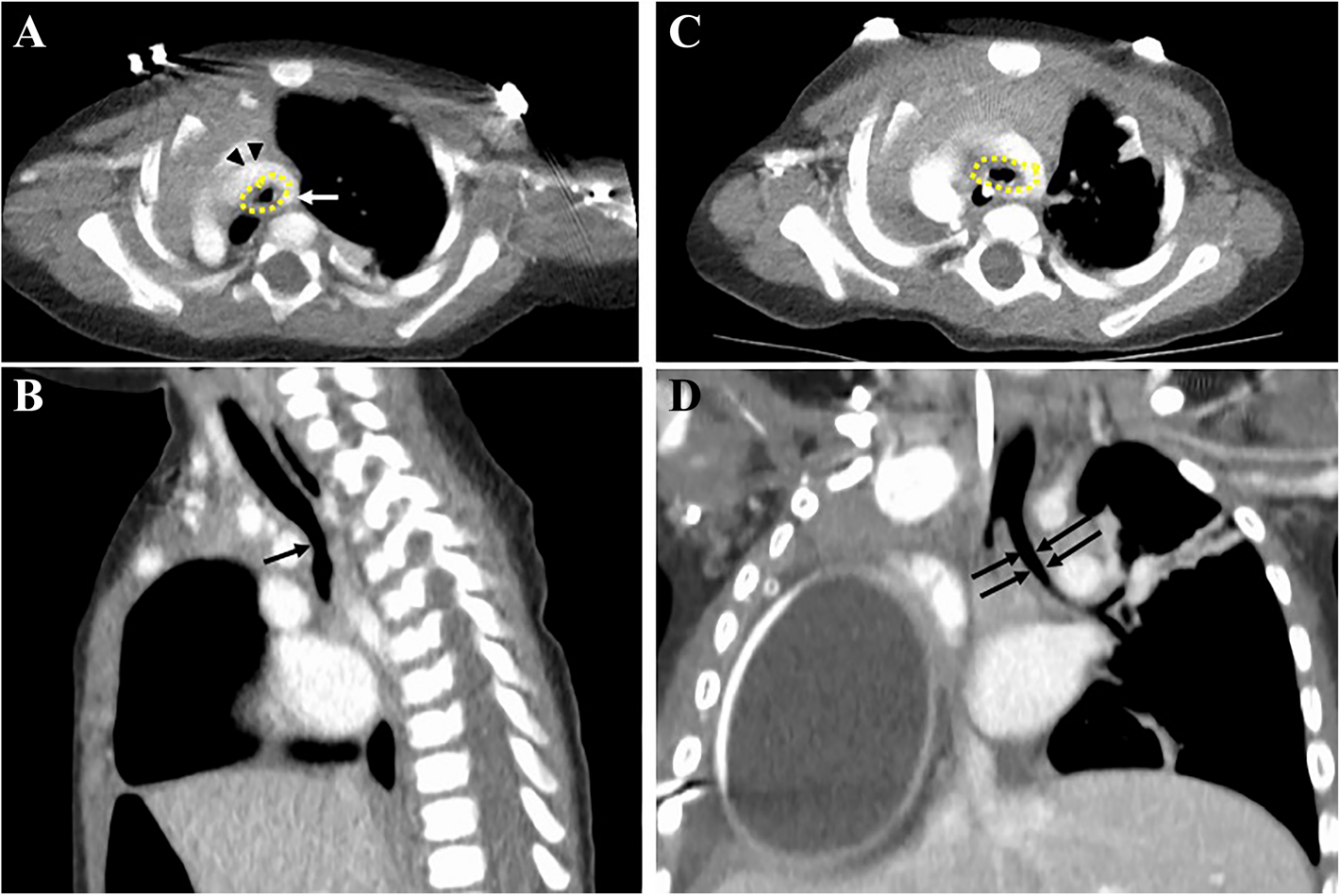
**a** A CT image shows a functional pseudo-vascular ring formed by the aorta (black arrow heads) and the aberrant right subclavian artery (white arrow) causing tracheal compression (dotted yellow outline). **b** A sagittal view of a CT image showing tracheomalacia and compression from the pseudo-vascular ring (black arrow). **c**, **d** A post-operative CT image after the placement of a tissue expander in the extrapleural space, showing the improvement of the tracheal compression (C: dotted outline and D: black arrows)

At 11 months old, the patient developed stridor and respiratory illnesses. Computerized tomography (CT) scan and bronchoscopy showed moderate-to-severe distal tracheomalacia with mainly anterior tracheal compression and slight posterior compression of her esophagus resulting from a pseudo-arterial ring formed by aortic arch and aberrant right subclavian artery (ARSA) (Fig. 1a, b). After a multi-disciplinary discussion with pediatric surgery, otolaryngology, congenital cardiac surgery, and vascular surgery, the consensus was that her tracheal compression was mainly caused by her aortic arch wrapping around the trachea as it shifted to the right chest. The posterior compression of her esophagus by the ARSA was deemed not significant at the time. She underwent a tissue expander placement (Integra®, PMT) with 80 ml of saline in the right extrapleural space through a right thoracotomy to push her heart leftward and anteriorly. Intraoperative bronchoscopy showed immediate improvement of her tracheomalacia (Fig. 1c, d).

**Fig. 2:**
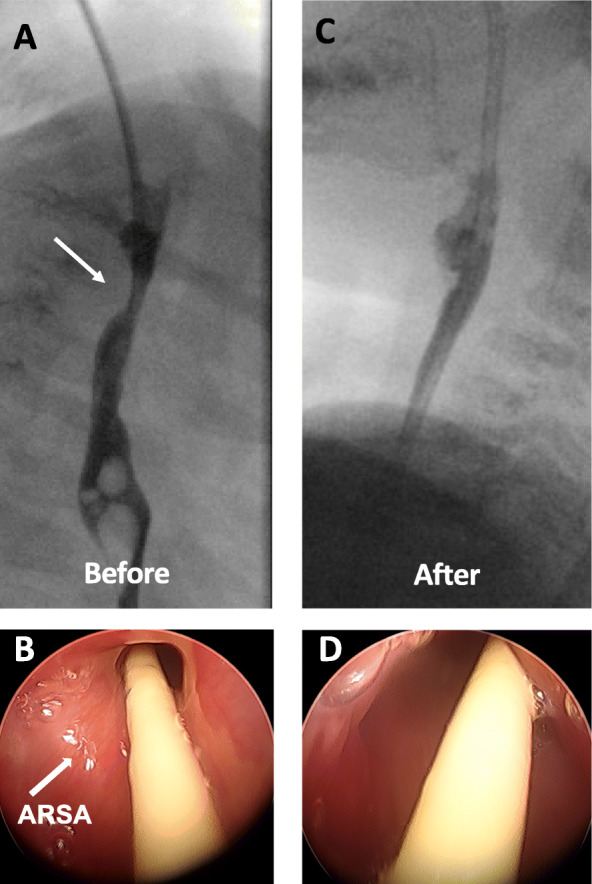
**a** Pre-operative esophagram shows posterior compression of the esophagus (white arrow) by ARSA. **b** Intraoperative esophagoscopy shows narrowing of the esophagus due to the posterior compression by ARSA (white arrow). **c**, **d** Resolution of posterior compression by the aberrant right subclavian artery (ARSA) after the ARSA transposition is seen on post-operative esophagram (**c**) and intraoperative ridged esophagoscopy (**d**)

On postoperative day (POD) 2, she developed progressive dysphagia after being advanced to regular diet. An esophagram showed worsening posterior compression of the esophagus by the ARSA, consistent with dysphagia lusoria (Fig. 2a, b). A nasogastric feeding tube was placed for temporary feeding and she underwent semi-elective transposition of the aberrant right subclavian artery to the right common carotid through a supraclavicular approach. She recovered without complications and her dysphagia lusoria resolved (Fig. 2c, d). The patient is developing well overall but has had recurrent symptoms of tracheomalacia at 14 months and 24 months of age, both of which resolved with additions of 15 ml and 20 ml of saline to her tissue expander, respectively. At 30 months of age, she outgrew her tissue expander and underwent a successful replacement with a larger one.

## Discussion and conclusions

We report here a case of an infant girl who was born with right-sided CSL and ARSA, along with other congenital anomalies, who underwent extrapleural tissue expander placement at 11 months for tracheal compression. We believe that due to her posterior compression of her esophagus by the ARSA, she developed dysphagia lusoria requiring an ARSA transposition. To our knowledge, this is the first report of this occurrence. Of the many anomalies that could exist with CSL, there has only been one case report of a patient with concurrent ARSA [5]. Although there are literature available on separate management of ARSA and CSL, this rare combination presented a unique challenge – whether to address both anomalies at the same time or to address one at a time, and if so, which one should be addressed first. This led us to pursue a multi-disciplinary, step-wise approach to her management. In an adult, compression due to acquired single lung is often managed by placement of a tissue expander to alleviate the mediastinal shift [6]. In children, silicone implants with or without aortopexy as well as tracheal reconstruction and diaphragmatic translocation to correct the mediastinal shift secondary to CSL have been used [1, 3, 7–11].

The most common repair of symptomatic ARSA is ligation of ARSA near the aortic arch followed by reimplantation, usually to the ipsilateral common carotid artery to avoid subclavian ischemia or steal syndrome and maintain perfusion of the (usually dominant) hand [12]. Supraclavicular, median sternotomy and both right and left thoracotomy approaches have been described [12]. In adults, combined open and endovascular approaches using endoluminal occlusion of ARSA followed by transposition have been also used [5, 13].

It can be argued that our patient’s initial symptoms from tracheal compression could have been due to anatomic consequences of the ARSA, and ligation and reimplantation of ARSA should have been performed before considering tissue expander placement to reduce the number of operations and encourage contralateral pulmonary hyperplasia. However, as our patient grew, more volume needed to be added to the tissue expander and eventually replaced to a larger one due to recurrence in respiratory symptoms. This strongly suggests that ARSA reimplantation alone would have not addressed tracheal compression secondary to mediastinal rotation from CSL and supports our step-wise approach. Of note, tracheoplasty was not considered for this patient as small trachea and bronchi make surgical tracheoplasty very challenging. Furthermore, the use of mesh, which is often used in conventional tracheoplasty can be problematic in the long term as it can cause airway stricture and excessive scars as the patient continues to grow.

Prompt work-up involving a multidisciplinary team is crucial for surgical planning and treatment of rare congenital single lung and symptomatic aberrant subclavian artery. Overall, we approached this complex patient in step-wise manner and were able to recognize and address her tracheomalacia and dysphagia lusoria promptly.

## Data Availability

Not applicable.

## Abbreviations

ARSA: Aberrant right subclavian artery
CSL: Congenital single lung
DOL: Day of life
POD: Post-operative day
RSA: Right subclavian artery
TEF: Tracheoesophageal fistula
